# Progression From Moderate to Severe Hypoxic-Ischaemic Injury May Not Affect Neuropsychological Responses in Rats, as Suggested by Psycho-Behavioural Tests

**DOI:** 10.1192/bjo.2026.11210

**Published:** 2026-06-30

**Authors:** Paola Cantalapiedra, Marc Chillida, Jon Ander Alart, Dra Ana Catalan, Daniel Alonso-Alconada

**Affiliations:** 1 University of the Basque Country, Leioa, Spain; 2 OSI Bilbao-Basurto, Bilbao, Spain

## Abstract

**Aims:**

Hypoxia-ischaemia (HI) is one of the leading causes of neurological injury and death among newborns. In developed countries, its incidence is estimated in approximately 1/1000 neonates, while globally it can represent up to 23% of children’s mortality rate. In those who survive, long-term neuropsychological impairments may emerge as a consequence of HI, causing a significant impact in quality of life.

**Objectives:**

Using a preclinical model of neonatal HI, the aim of this study was to investigate whether a progression in HI severity (from moderate to severe) may be associated with worsened functional and psycho-behavioural performance.

**Methods:**

Seven-day-old (P7) Sprague Dawley neonatal rats were randomly assigned to: i) HI-moderate (left common carotid artery ligation + 120 min of 8%O_2_/92%N_2_ hypoxia; n=13); ii) HI-severe (same surgical procedure and 150min of hypoxia; n=18); Sham (without HI, n=36). At P42 (early young equivalent) and P90 (adult), animals underwent three behavioural tests: the novel object recognition (NOR) test to analyse cognitive deficits in recognition memory, the cylinder test to measure sensorimotor asymmetries and deficits, and the T-maze test to evaluate possible cognitive disabilities.

**Results:**

When evaluating cognitive deficits in recognition memory with NOR test, both moderate (p<0.01) and severe (p<0.05) injured animals showed worse performance than sham at P42. At P90, this was also observed in moderate-injured animals (p<0.001). In the cylinder test, HI-moderate (p<0.01) and HI-severe (p<0.001) groups showed significant sensorimotor asymmetries when compared to sham at P42 and P90, which were independent of HI severity. Similarly, all HI-animals revealed a reduction in alternation in the T-maze test, thus suggesting cognitive disabilities in both moderate (p<0.05) and severe (p<0.0001)models vs sham. Again, no differences were found when comparing severities.

**Conclusion:**

Psycho-behavioural assessments showed the development of cognitive deficits and neuromotor impairments in the long term after neonatal HI. Further studies may confirm if the absence of differences between moderate and severe HI models may relate to a critical threshold reached in the former and/or to possible compensatory mechanisms in the latter.

